# Acute Central Toxic Keratopathy Induced by Exposure to Chinese Herbal Medicine Fluid for Verruca Plana: A Case Report

**DOI:** 10.1155/crop/8657147

**Published:** 2025-07-22

**Authors:** Shuang Zhang, Yong Tao

**Affiliations:** Department of Ophthalmology, Beijing Chao-Yang Hospital, Capital Medical University, Beijing, China

**Keywords:** central toxic keratopathy, Chinese herbal medicine, steroid treatment, verruca plana

## Abstract

**Purpose:** The purpose of this study was to report a case of acute central toxic keratopathy due to exposure to Chinese herbal medicine fluid treating verruca plana.

**Methods:** A 46-year-old woman presented with pain and blurred vision in her right eye for 3 days. Her right eye was unintentionally exposed to a medication in liquid form treating the verruca plana on her eyelids. The drug was a compound preparation with complex Chinese herbal medicinal ingredients.

**Results:** Slit lamp examination showed central diffuse corneal subepithelial haze with granular shapes and anterior stromal opacity. Corresponding with her clinical manifestations, anterior segment optical coherence tomography revealed diffuse abnormal highly reflective signal in the anterior stroma within 349 *μ*m and in vivo confocal microscopy found inflammatory infiltration in the subepithelial and the anterior stromal layer. Thus, tobramycin dexamethasone eye drops and artificial tears were prescribed for her, which proved effective. Her clinical symptoms and signs were both resolved after steroid treatment and remained stable at the 1-month follow-up.

**Conclusion:** Acute central toxic keratopathy could occur after exposure to Chinese herbal medicine fluid, and enhanced topical steroid treatment worked well for alleviating inflammation and reducing corneal opacity.

## 1. Introduction

Toxic keratopathy (TK) could lead to corneal damage and dysfunction, which usually is induced by exposure to ophthalmic medications or other toxic substances. The clinical manifestations of TK are complicated, ranging from mild punctate keratitis to severe or ring ulcerative keratitis [1]. Previous studies report TK is caused by various reasons such as topical use of expired eye drops [2], anesthetics [3, 4], antiseptics [5], and sea water [6]. The ocular manifestations in different cases vary from one to another. In this study, we present a rare case of central toxic keratopathy (CTK) due to topical contact with Chinese herbal medicine fluid treating verruca plana, and written informed consent was obtained from the patient. Although CTK characterized by central corneal opacity usually occurs after laser refractive surgery or contact lens wear [7], similar manifestations were noted in our case. Instead of epithelial lesions, this patient manifested as central diffuse subepithelial haze and anterior stromal opacity with intact epithelium. Moreover, we performed anterior segment optical coherence tomography (AS-OCT) and in vivo confocal microscopy (IVCM) examinations on this patient, the results of which were enlightening.

## 2. Case Presentation

**Figure 1:**
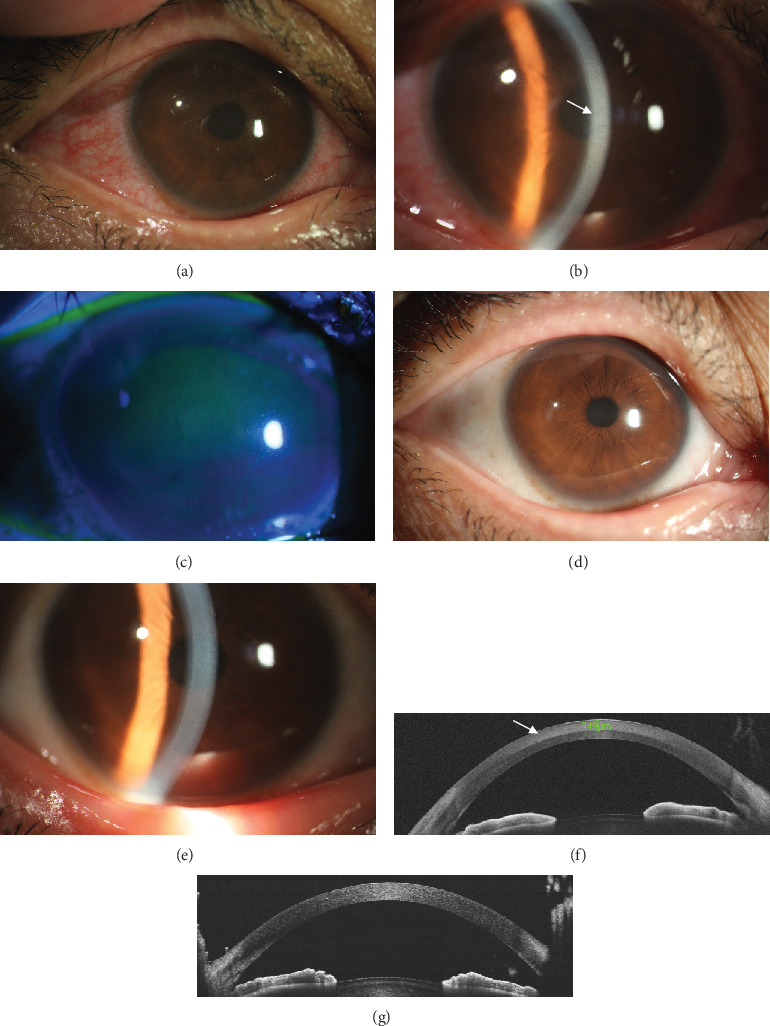
Slit lamp examination and anterior segment optical coherence tomography (AS-OCT) of the patient's right eye. (a) Conjunctival mixed congestion during the first visit. (b) Central diffuse corneal subepithelial haze with granular shapes and anterior stromal opacity (white arrow). (c) Fluorescein staining of the patient's right eye during her first visit. (d) The conjunctival congestion was relieved completely after 1-week treatment of topical steroid. (e) The subepithelial haze and anterior stromal opacity were largely alleviated. (f) AS-OCT examination showed a highly reflective signal from 0 to 349 *μ*m of the cornea corresponding to the anterior stroma. (g) The highly reflective signal in the anterior stroma disappeared after 1-week treatment.

**Figure 2:**
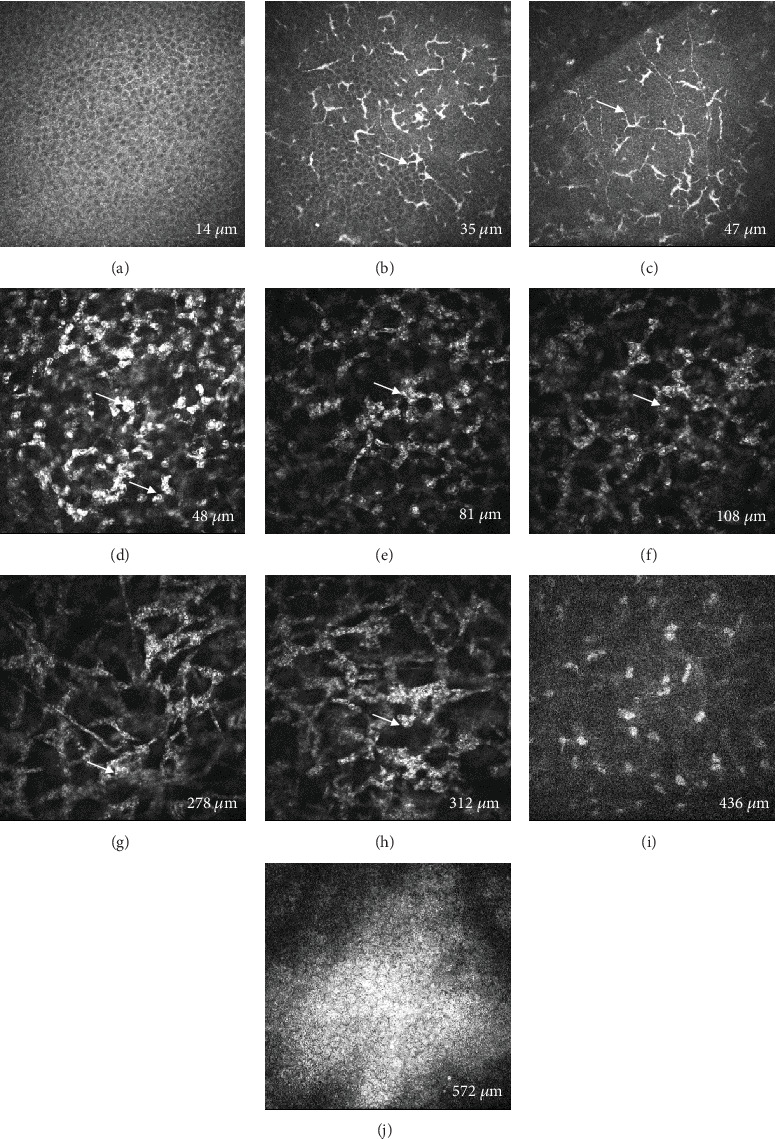
In vivo confocal microscopy examination of the patient during her first visit (400 × 400* μ*m). (a) Normal corneal epithelium. (b, c) Dendritic cell infiltration (white arrows) in the subepithelial layer. (d–h) Stromal cell swelling with inflammatory cell infiltration (white arrows). (i) Normal stromal cells in the posterior stromal layer. (j) Normal corneal endothelial cells.

A 46-year-old woman presented to our hospital with pain and blurred vision in her right eye for 3 days. She mentioned that she once smeared a medication in liquid form treating verruca plana around the skin near her right eye, which was not prescribed by the doctor and self-administered. Subsequently, some fluid accidentally entered her eye. The drug was a compound preparation over the counter with complex Chinese herbal medicinal ingredients including *Cyclobalanopsis*, *Lithospermum*, *Lonicera*, Berberidaceae, *Sophora*, Rutaceae, podophyllotoxins, and *Aloe*. The best-corrected visual acuity (BCVA) in her right eye reduced to 20/40, and the intraocular pressure (IOP) was normal with 15 mmHg. Slit lamp examination revealed conjunctival mixed congestion (Figure 1a), central diffuse corneal subepithelial haze with granular shapes, and anterior stromal opacity (Figure 1b). However, fluorescein staining was negative with no epithelial defect in her right eye (Figure 1c). She then underwent AS-OCT and IVCM examinations. AS-OCT showed diffuse abnormal highly reflective signal in the anterior stroma from 0 to 349-*μ*m thickness of the cornea (Figure 1f). IVCM found the superficial corneal epithelium was intact with normal appearance (Figure 2a) while the infiltration of dendritic cells in the subepithelial layer (Figure 2b,c) and stromal cell swelling with inflammatory cell infiltration (Figures 2d, 2e, 2f, 2g, and 2h) in the anterior stromal layer were noted, which was consistent with the results of AS-OCT. The posterior stroma and corneal endothelial cells remained normal without obvious lesions (Figure 2i,j). Considering the remarkable inflammation in the subepithelial and anterior stromal layer, she was prescribed tobramycin dexamethasone eye drops four times a day and artificial tears four to six times a day. One week later, she came back with an increase of BCVA to 20/20. The conjunctival mixed congestion was completely resolved (Figure 1d), and the corneal subepithelial haze and anterior stromal opacity were also largely alleviated (Figure 1e). The IOP remained normal with 14 mmHg. Besides, corresponding with the ocular examination, AS-OCT showed the highly reflective signal in the anterior stroma disappeared (Figure 1g). Then, the patient experienced tapering of the steroid eye drops for the next 3 weeks. At the 1-month follow-up, the patient's right eye was stable with the BCVA 20/20, and there were slightly granular changes in the anterior stroma on her right eye with no deterioration.

## 3. Discussion

TK is usually characterized by corneal epithelial lesions, and in more serious cases, ulcerative keratitis and ring corneal keratitis would be noted [1]. The possible factors contributing to TK could be exposure to different eye drops, associated preservatives, or a variety of chemicals. In this study, we reported a rare case of CTK secondary to eye contact with Chinese herbal medicine fluid. The herbal drug contained complex ingredients, which were used to corrode verruca plana. Hence, the herbal drug caused toxic damage to the cornea. The patient's clinical manifestations were diffuse corneal subepithelial haze with granular changes and anterior stromal opacity with intact epithelium. However, previous studies [2, 3, 5, 8–10] reported that similar TK caused by other factors usually started with epithelial defect and progressed with more severe complications. For instance, exposure to the alcohol-containing antiseptics could lead to diffuse corneal epithelial defect, necrosis of adjacent conjunctiva, and corneal opacity [5, 9]. Huda et al. [2] described two cases of TK following instillation of expired topical eye drops, which were characterized by diffuse superficial punctate epithelial defects as well. In addition, Mehrdad et al. [8] reported one patient who experienced inadvertent exposure to topical minoxidil 5% solution also developed corneal epithelial irregularity and subsequently corneal thinning and opacity. On the contrary, the patient in this study presented with the negative result of fluorescein staining, although the drug should have damaged the epithelium initially. It was on the third day that she referred to our hospital after exposure to the herbal drug, which might account for the repair of the initial epithelial lesion during this period. In terms of the intact corneal epithelial layer and central anterior stromal opacity noted in this case, the clinical signs resembled CTK reported by previous literature to some extent. CTK is characterized by noninflammatory, anterior to midstromal, central corneal opacification accompanied by significant corneal flattening after laser refractive surgery or contact lens use [7]. With the progression and remission of CTK, hyperopic shift usually occurs due to the associated corneal flattening and anterior corneal curvature change. Although no obvious corneal flattening was found by slit lamp examination during the inflammation and after resolution, we did not perform corneal topography examination on this patient to confirm this, which was one of the limitations of this study.

In this case, the herbal drug mainly caused an immune reaction characterized by inflammatory infiltration in the subepithelial and anterior stromal layers, as demonstrated by IVCM. Previous studies [1, 11] reported the IVCM features of TK, including lower basal cell density, lower corneal nerve fiber length, greater dendritic cell density, and greater dendritic size. Similarly, we noted increased dendritic cell density and size in the subepithelial layer. The above evidence supported the following enhanced topical steroid treatment for the patient, which proved effective. Therefore, AS-OCT examination and IVCM were both useful for helping diagnosis and guiding treatment. When inflammatory infiltration was found by IVCM, enhanced steroid treatment should be used to control the overreactive immune response.

## Data Availability

Data sharing is not applicable to this article as no new data were created or analyzed in this study.

## Nomenclature

TK: toxic keratopathy
CTK: central toxic keratopathy
AS-OCT: anterior segment optical coherence tomography
IVCM: in vivo confocal microscopy
BCVA: best-corrected visual acuity
IOP: intraocular pressure
