# Supplementary material for: Acute Central Toxic Keratopathy Induced by Exposure to Chinese Herbal Medicine Fluid for Verruca Plana: A Case Report
Source: Case Rep Ophthalmol Med. 2025 Jul 22;2025:8657147. doi: 10.1155/crop/8657147 (PMC12308051; doi:10.1155/crop/8657147)
Supplement: Supporting Information — Additional supporting information can be found online in the Supporting Information section. [file 8657147.f1.pdf]

Dear Editor,

We submit our manuscript entitled " **Acute Central Toxic Keratopathy Induced by Exposure to Chinese Herbal Medicine Fluid for Verruca Plana: A Case Report** " to ***Case reports in ophthalmological medicine*** for publication.

This is a case-report study about a patient who developed acute central toxic keratopathy resulting from exposure to Chinese herbal medicine fluid treating verruca plana. In general, the toxic keratopathy is characterized by corneal epithelial lesions such as mild punctate keratitis or severe ulcerative keratitis. Instead, this patient manifested as diffuse corneal subepithelial haze with granular shapes and anterior stromal opacity with intact epithelium. Therefore, the complicated Chinese herbal ingredients mainly caused immune reaction in this patient's cornea. Corresponding with her clinical manifestations, anterior-segment optical coherence tomography revealed diffuse abnormal highly reflective signal in the anterior stroma within 349μm and in vivo confocal microscopy found inflammatory infiltration in the subepithelial and the anterior stromal layer. Thus, enhanced topical steroid treatment was prescribed for her, which turned out to be effective. Her clinical symptoms and signs were both resolved after steroid treatment and remained stable at the one-month follow-up. From this patient, we drew a conclusion that acute central toxic keratopathy could occur after exposure to Chinese herbal medicine fluid and enhanced topical steroid treatment worked well for alleviating inflammation and reducing corneal opacity.

The work described has not been submitted elsewhere for publication in similar forms.

Kind regards.

Your sincerely,

Dr. Shuang Zhang, MD;

Email: zshuang95@qq.com

Department of Ophthalmology, Beijing Chao-Yang Hospital, Capital Medical University

No. 8, Gongyinan Road, Chaoyang District

Beijing 100020, P.R China

Tel.: +86 10 85231717
